# Supplementary material for: Anti-Tumor Effects of Cecropin A and Drosocin Incorporated into Macrophage-like Cells Against Hematopoietic Tumors in Drosophila mxc Mutants
Source: Cells. 2025 Mar 7;14(6):389. doi: 10.3390/cells14060389 (PMC11940895; doi:10.3390/cells14060389)
Supplement: Supplementary file 1 [file cells-14-00389-s001.zip › cells-3480607-supplementary.pdf]

Appendix

Supplementary Figure legends

Figure S1. Immunostaining and quantification of apoptosis in wing imaginal discs of larvae harboring fat body-specific induction of *Dro* or *CecA1* overexpression.

(a-f) Immunostaining of wing imaginal discs from the third instar larvae with anti-cDcp1 antibody. Wing discs from normal control larvae (*w/Y; r4*>+)(a), those from control larvae with ectopic expression of *Dro* (*w/Y; r4>Dro*) (b) or *CecA1* (*w/Y; r4>CecA1*) (c) in the fat body are shown. Blue indicates DNA staining, and green indicates anti-cDcp1 immunostaining signals. Scale bars: 100 μm.

Figure S2. Immunostaining and quantification of cell proliferation in LGs of mxc^{mbn1} larvae with a fat body-specific expression of *Dro* or *CecA1*.

(a-f) Immunostaining images of LGs stained anti-PH3 antibody. LGs from (a) normal control larvae (no fat body-specific induced expression) (*w/Y; r4*>+), (b) LGs with ectopic expression of *Dro* (*w/Y; r4>Dro*), or (c) *CecA1* (*w/Y; r4>CecA1*) in the FB are shown. LGs from (d) mxc^{mbn1} larvae without the ectopic expression (mxc^{mbn1}*/Y; r4*>+), (e) LGs harboring the fat body-specific expression of *Dro* (mxc^{mbn1}*/Y; r4>Dro*) or (f) *CecA1* (mxc^{mbn1}*/Y; r4>CecA1*) are shown. Blue in a-f indicates DNA staining, and green in a-d (white in a′-f′) indicates anti-PH3 immunostaining signals. Scale bars: 100 μm. (g) The graphs indicate the percentage of M-phase cells in the anterior lobe regions in the right or left hemispheres of LGs from the larvae with the ectopic expression of *Dro* and *CecA1*. Significant differences between the experimental groups were determined using one-way ANOVA multiple comparisons (****$p < 0.0001$, ns: not significant). The red line indicates the mean value of the percentage, and the error bars indicate SEM.

Figure S3. Absence of the *Drs* gene transcription in hemocytes, as confirmed by the *Drs-YFP* reporter in mxc^{mbn1} larvae.

(a, b) Fluorescence microscopic images of the circulating hemocytes in the third instar mature larva carrying the *Drs-YFP* reporter, which can monitor the transcription of the *Drs* gene by a YFP fluorescence (a′, b′). Blue: DNA staining with DAPI. Green in a, b (white in a′, b′): a YFP fluorescence. Hemocytes from normal control (*w/Y*) (a) and mxc^{mbn1} (mxc^{mbn1}*/Y*) (b) larvae. Note that no YFP fluorescence exceeding the background level is detected (n > 200 hemocytes from 5

larvae were examined). Scale bars: 10 μm. (c) A positive control to confirm the expression of *Drs-YFP* reporter in the FB of *mxc*^{mbn1} larvae. Scale bars: 100 μm.

Figure S1

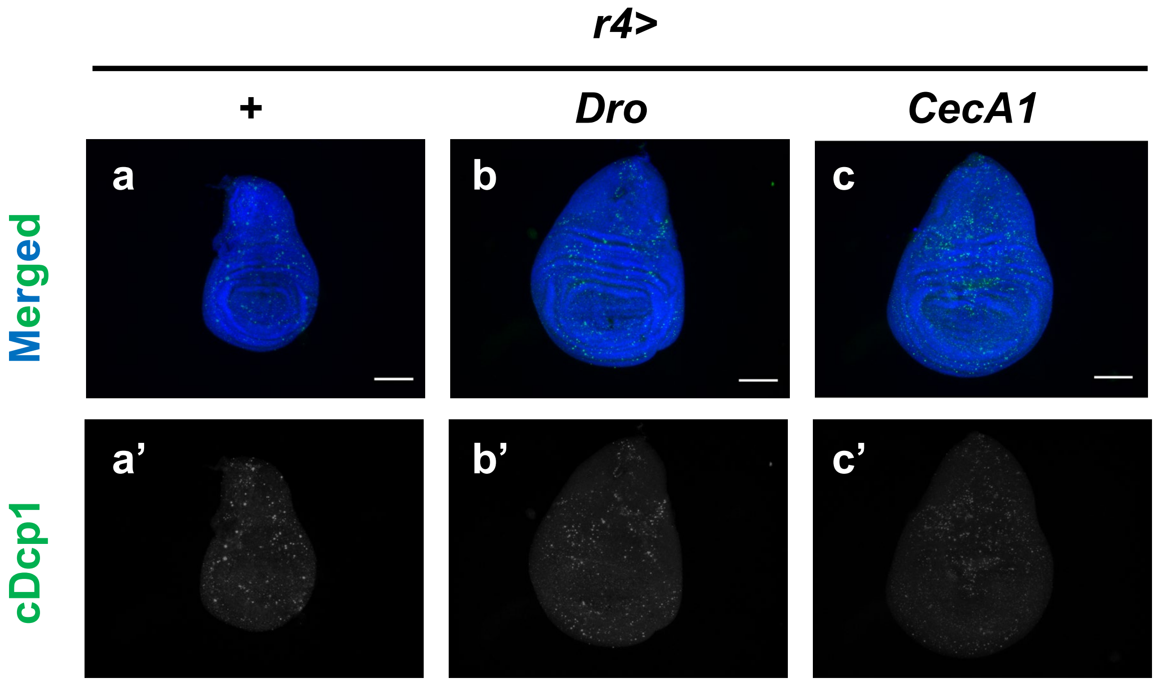

Figure S2

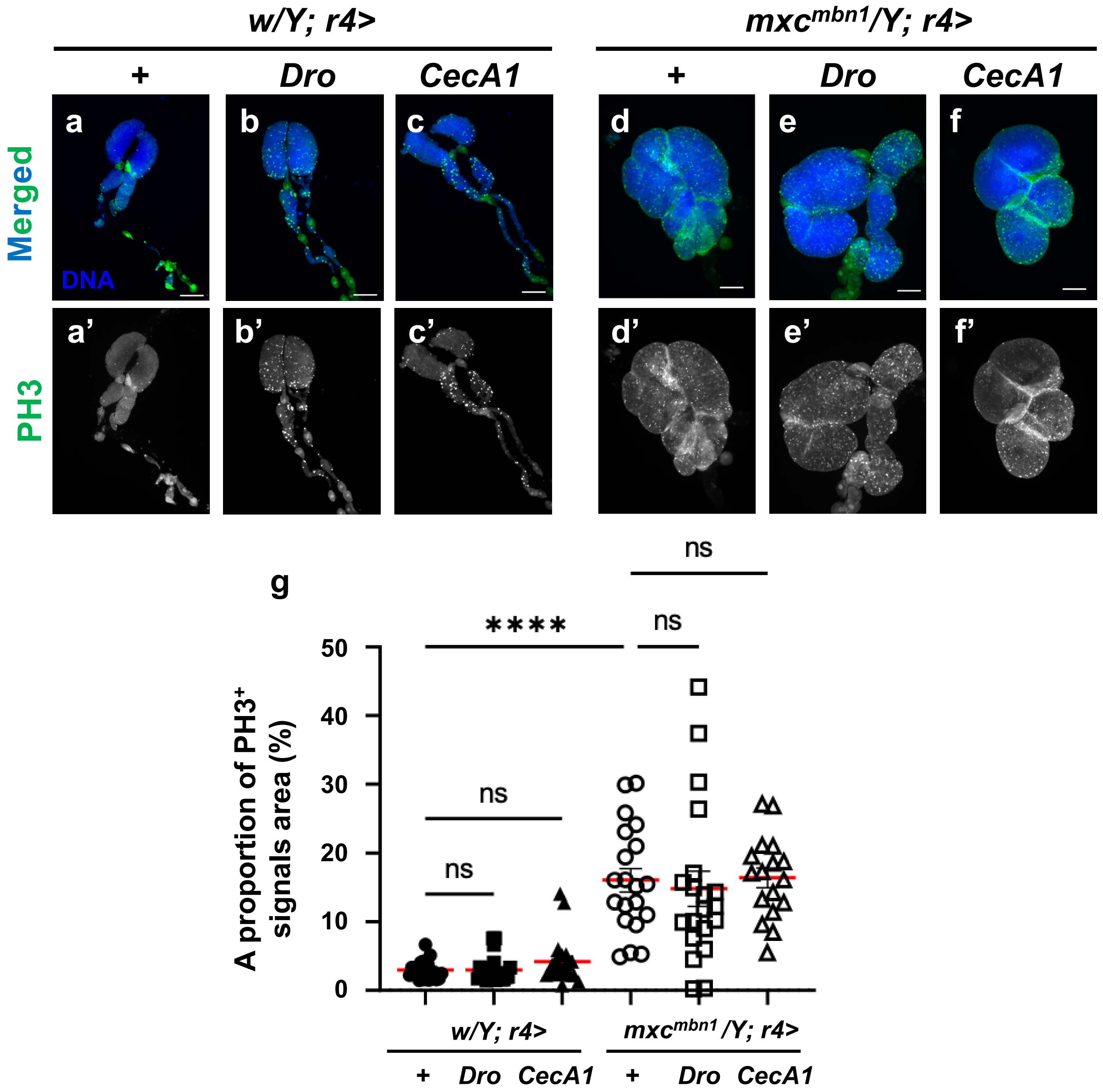

w/Y; r4>
mxc^mbn1/Y; r4>
+
Dro
CecA1
+
Dro
CecA1
a
b
c
d
e
f
Merged
DNA
a'
b'
c'
d'
e'
f'
PH3
g
A proportion of PH3+ signals area (%)
50
40
30
20
10
0
****
ns
ns
ns
ns
w/Y; r4>
mxc^mbn1 /Y; r4>
+
Dro
CecA1
+
Dro
CecA1

Figure S3

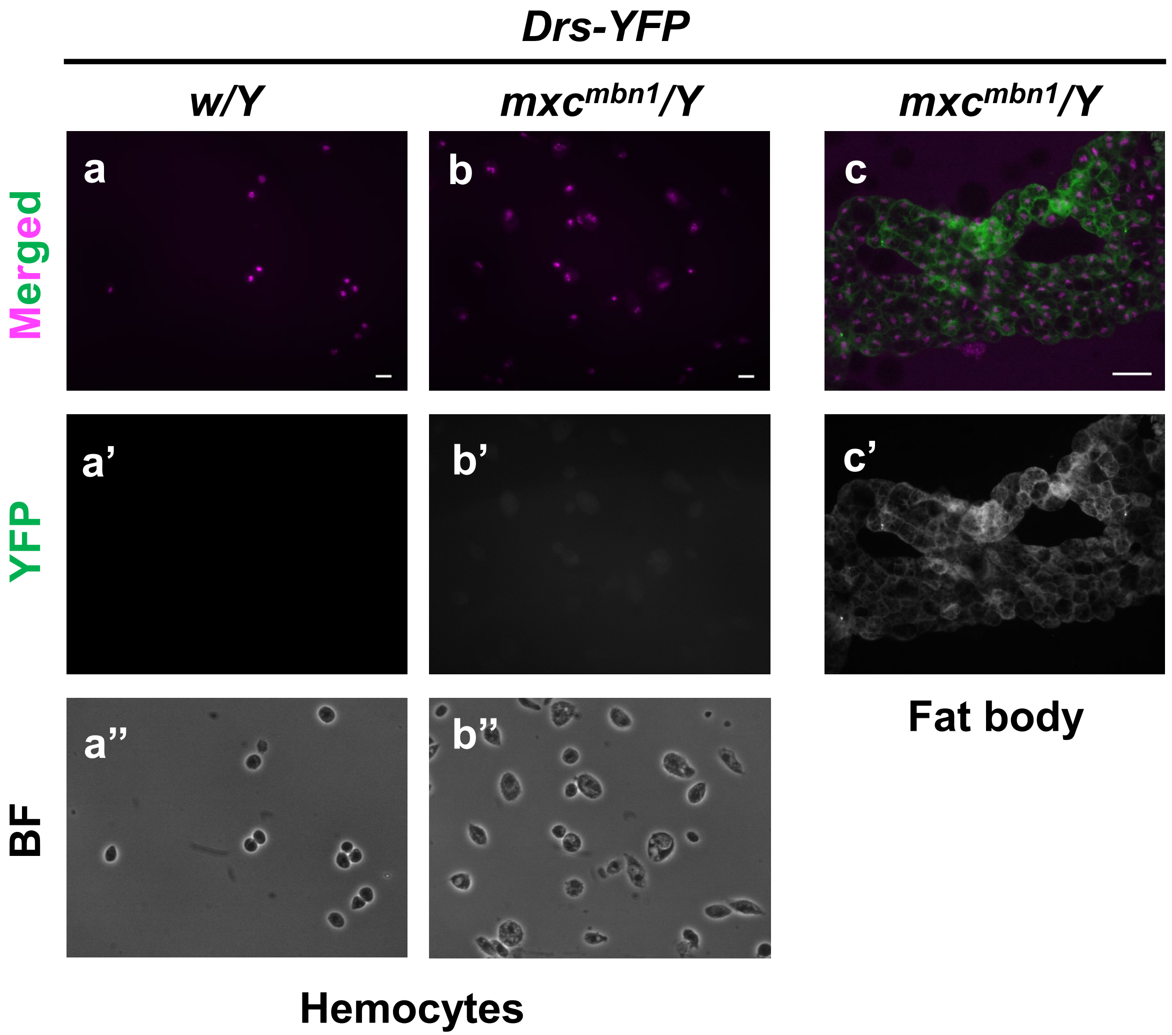

Drs-YFP
w/Y
mxc mbn1/Y
mxc mbn1/Y
Merged
YFP
BF
a
b
c
a'
b'
c'
a''
b''
Fat body
Hemocytes
